# Mutual research capacity strengthening: a qualitative study of two-way partnerships in public health research

**DOI:** 10.1186/1475-9276-11-79

**Published:** 2012-12-18

**Authors:** Michelle Redman-MacLaren, David J MacLaren, Humpress Harrington, Rowena Asugeni, Relmah Timothy-Harrington, Esau Kekeubata, Richard Speare

**Affiliations:** 1 School of Medicine and Dentistry, James Cook University, Cairns, Australia; 2 School of Public Health, Tropical Medicine and Rehabilitation Sciences, James Cook University, Cairns, Australia; 3 Atoifi College of Nursing, East Kwaio, Malaita, Solomon Islands; 4 Atoifi Adventist Hospital, East Kwaio, Malaita, Solomon Islands; 5 Community Chief, East Kwaio, Malaita, Solomon Islands; 6 School of Public Health, Tropical Medicine and Rehabilitation Sciences, James Cook University, Townsville, Australia; 7 Tropical Health Solutions, Townsville, Australia

**Keywords:** Capacity building, Research capacity strengthening, Health research, Solomon Islands, Atoifi Adventist Hospital, Mutuality

## Abstract

**Introduction:**

Capacity building has been employed in international health and development sectors to describe the process of ‘experts’ from more resourced countries training people in less resourced countries. Hence the concept has an implicit power imbalance based on ‘expert’ knowledge. In 2011, a health research strengthening workshop was undertaken at Atoifi Adventist Hospital, Solomon Islands to further strengthen research skills of the Hospital and College of Nursing staff and East Kwaio community leaders through partnering in practical research projects. The workshop was based on participatory research frameworks underpinned by decolonising methodologies, which sought to challenge historical power imbalances and inequities. Our research question was, “Is research capacity strengthening a two-way process?”

**Methods:**

In this qualitative study, five Solomon Islanders and five Australians each responded to four open-ended questions about their experience of the research capacity strengthening workshop and activities: five chose face to face interview, five chose to provide written responses. Written responses and interview transcripts were inductively analysed in *NVivo 9.*

**Results:**

Six major themes emerged. These were: Respectful relationships; Increased knowledge and experience with research process; Participation at all stages in the research process; Contribution to public health action; Support and sustain research opportunities; and Managing challenges of capacity strengthening. All researchers identified benefits for themselves, their institution and/or community, regardless of their role or country of origin, indicating that the capacity strengthening had been a two-way process.

**Conclusions:**

The flexible and responsive process we used to strengthen research capacity was identified as mutually beneficial. Using community-based participatory frameworks underpinned by decolonising methodologies is assisting to redress historical power imbalances and inequities and is helping to sustain the initial steps taken to establish a local research agenda at Atoifi Hospital. It is our experience that embedding mutuality throughout the research capacity strengthening process has had great benefit and may also benefit researchers from more resourced and less resourced countries wanting to partner in research capacity strengthening activities.

## Introduction

Health research capacity strengthening is critical to improving health equity in less resourced countries [1]. In the past, capacity building was provided in health and development sectors to deliver training and/or distribution of materials [2]. However, training for individuals alone is inadequate to achieve the goal of improving health equity - institutional strengthening is also a key component of capacity building [3]. Research capacity building requires “the ongoing process of empowering individuals, institutions, organizations and nations to: define and prioritize problems systematically; develop and scientifically evaluate appropriate solutions; and share and apply the knowledge generated” [4]. The increased use of terminology such as *capacity development* and *capacity strengthening* in health and development reflects a change in the way we understand and operationalize this work. Capacity strengthening considers power imbalances (with power taken to mean the ability to direct or influence the behaviour of individuals and groups), cultural contexts, relationships between less resourced and more resourced, system requirements and the existing strengths of people participating in the capacity development process [5-11]. In the current literature about research capacity strengthening (RCS), the process appears to be largely one way with no guidelines on how the “experts”, mostly from more resourced countries can learn from those being “strengthened”, mostly in less resourced countries [2]. In this paper we use the term *capacity strengthening* instead of *capacity building* because *capacity strengthening* more accurately describes the mutual development of our research capacity as established researchers (researchers well established in their career with a sustained track record of leading research and publishing in peer-reviewed literature), emerging researchers (researchers who are pre-doctoral or early career researchers) and community chiefs supporting research.

In September 2009 a one-week introduction to health research workshop was facilitated at Atoifi Adventist Hospital, located in Solomon Islands’ most populated island, Malaita. This was a major event in the evolution of health research at the hospital and the surrounding communities. Atoifi Adventist Hospital is a 90 bed general hospital established by the Seventh-day Adventist church in 1966, 22 years prior to Solomon Islands independence from Great Britain. It is located on the remote east coast, and directly serves people who live a rural subsistence lifestyle in the East Kwaio language group and indirectly the people of the other 10 language groups on Malaita. There is a wharf and grass airstrip at the hospital but no roads. People access the hospital by walking (some for several days), by canoe, irregular shipping or twice weekly light aircraft. Health services at the hospital have been characterised by the legacy of colonisation, Christianisation and western biomedical dominance that has taken little account of local customs, practices and beliefs. This is most starkly experienced by several thousand people who live in the mountainous interior of the island who have chosen local social, cultural and spiritual autonomy over introduced colonial and Christian practices [12-16]. Atoifi College of Nursing is located on the hospital campus and educates half of the nursing workforce for the Solomon Islands.

Research has not been a priority for the hospital or college of nursing in its 40 year history. However there has been periodic health research that has taken place at the hospital and surrounding community. Two hospital based studies have been published, one in 1984 on birthweight at Atoifi using birthweight records [17] and the second in 2000 on the intravenous use of coconut water [18]. Both of these were led and published by visiting international doctors and not a part of ongoing RCS activities.

Having worked as a medical scientist at the hospital from 1992–1994, DM returned in 1999 to conduct public health research, initially for masters research, and from 2001 for doctoral research at the hospital and surrounding communities [14,19,20]. DM embedded collaborative processes throughout his research including design, analysis and presentation of results with village health worker and East Kwaio chief, EK [21,22]. Since then DM, MRM and others have supported Solomon Islander colleagues in their initial steps to include research in their professional practice. HH, a Solomon Islander, is the principal of the College of Nursing and conducted a study at Atoifi hospital in 2003 for a Master of Adult Education qualification. Having worked together during 1992–1994 and conducting master and doctoral research at similar times both HH and DM have had ongoing discussion about the need to strengthen research capacity at Atoifi [23]. The two colleges of nursing in Solomon Islands provide only a brief introduction to research in undergraduate courses. Therefore, access to substantial research training is very difficult, and only small numbers of students are able to access scholarships to international universities. On their return to Solomon Islands, most of these people hold positions in the nation’s capital with very few willing or able to work, or conduct research, in provincial locations such as Atoifi. The nation has hosted many international health researchers. However, most of these researchers work with Solomon Islands Medical Training and Research Institute, located in the capital, Honiara. This is despite the majority of the population and greatest burden of disease being located in rural provincial villages across the island archipelago. To enable RCS at Atoifi different models were explored, and ultimately departed from the model that selects an elite few for international universities, to one the engages with provincial institutions and local grassroots communities. This more participative model allowed for a broad collection of local health professionals and community leaders to be a part of health research for the first time – and enabled local research questions to be developed, local research methods adapted and local answers to be developed to inform policy and procedures to address local health issues.

In 2008, the Director of Nursing and the mental health nurse from Atoifi Adventist Hospital (AAH) and EK, with the assistance of DM, travelled to Australia to present on social and cultural issues faced by the new mental health service at the hospital at an international community mental health conference. During that visit a request was made to James Cook University (JCU) to assist in the next steps in increasing the research capacity of Hospital and College of Nursing staff. It was requested that the RCS approach be tailored to the unique context of the remote hospital with limited research infrastructure, its unique history and social, cultural and spiritual diversity. It was an aim for many at the hospital to be able to lead collaborative research teams in the future. Within this context, a group of three public health researchers from JCU Australia (including MRM and DM) travelled to Atoifi in September 2009 to conduct the one-week *Introduction to Health Research* workshop, the details of which are published elsewhere [24]. As a result of this workshop, HH, RA and RTH from Atoifi were offered and accepted adjunct appointments at JCU. These appointments enhanced opportunities for HH, RA and RTH to learn about research, lead research activities and support nursing colleagues and students and community members to undertake research. HH and RA travelled to Cairns, Australia to formally present on RCS at the 2010 Fulbright Symposium, providing opportunities to observe and discuss the nature of research, various research designs and results and to experience the accountability of the scientific community [25].

At the completion of the 2009 *Introduction to Health Research* workshop the same group of Solomon Islanders requested a further practical ‘learn-by-doing’ workshop that included systematically and rigorously conducting small studies into health issues of importance to local communities and health services. Specific research needs were identified by workshop participants from Solomon Islands to conduct public health studies that were scientifically rigorous and could inform local health policy and practice. In response, public health researchers from James Cook University, University of Tasmania and New South Wales Health, Australia partnered with the group to facilitate a two-week workshop in April 2011. Local participants not only included health professionals, but at the request of the Solomon Islander researchers on the team, included leaders from coastal and mountain communities, particularly chiefs, village headmen, pastors and teachers, both males and females. Some of these lay participants were illiterate in written language, but brought a wealth of social, cultural and religious knowledge and linkages with extensive community networks.

Learning outcomes for this subsequent two-week workshop addressed topics of research design, data collection and reporting with teaching strategies that included the planning, conduct and reporting of pilot studies on three topics: tuberculosis (TB); HIV; and intestinal parasitic worms. The research topics for the pilot studies were mutually identified by Atoifi staff and community leaders as important health issues in their community and ones in which the Australian team members had skills and experience. In addition, the need for a community survey for lymphatic filariasis (LF) was identified during the two weeks of the workshop when a 40 year old male presented to the hospital with elephantiasis of the lower leg. Responding to this clinical presentation a fourth pilot study, a LF survey, was planned by Solomon Islander and Australian members of the workshop team (RS is a public health researcher who helped establish the WHO collaborating Centre for Lymphatic Filariasis and Soil Transmitted Helminths at JCU). The survey was conducted by Solomon Islander researchers in the days after the Australian researchers had departed.

The structure of the research workshop was a mixture of formal lectures and extensive group interaction at every stage of planning, conducting and reporting the pilot studies. The outcomes were impressive: during 10 working days 43 participants designed four pilot studies, obtained local ethics approval, collected data, completed analysis and interpretation, and presented the findings in a local research symposium. Two weeks after the workshop, results from the TB study were presented at the Solomon Islands Annual National TB symposium. Results from all four pilot studies were also presented at the Inaugural Solomon Islands National Nursing Research Symposium in May 2012 and the TB pilot study has been published in peer-reviewed literature [26-30].

Participatory research frameworks [31,32] underpinned by decolonising methodologies [33-35] were used throughout the workshop. It was our experience that the workshop had appeared to be highly beneficial to all parties. We therefore conducted a systematic, reflective qualitative study with key workshop facilitators and participants to explore and document benefits experienced by both Australian and Solomon Islander researchers. We aimed to answer the research question: Was this research capacity strengthening activity a two-way, mutual process?

## Methods

A qualitative methodology was used to gather reflective responses on how the capacity strengthening had been experienced. Ten established researchers, emerging researchers and community chiefs who provided leadership for the workshop and/or pilot research activities participated. In this purposeful sample there were two East Kwaio chiefs and three emerging researchers from Atoifi Hospital/College of Nursing (five of 43 Solomon Islander workshop participants). All five researchers from Australian universities and health institutions participated. The group consisted of seven males (3 Solomon Islander; 4 Australian) and three females (2 Solomon Islander; 1 Australian). Utilising qualitative methods allowed respondents from a wide variety of social, cultural, epistemological, educational, professional and linguistic backgrounds to reflect and respond to questions on their own experience. Four deliberately open-ended questions were posed: (i) When considering your capacity to undertake research, what have been the benefits for you (researcher/person), your organisation/community or more broadly? (ii) Were there any negatives? (iii) Do you have a story which demonstrates your experience of this capacity building? (iv) What do you think will happen next? Why?

**Figure 1:**
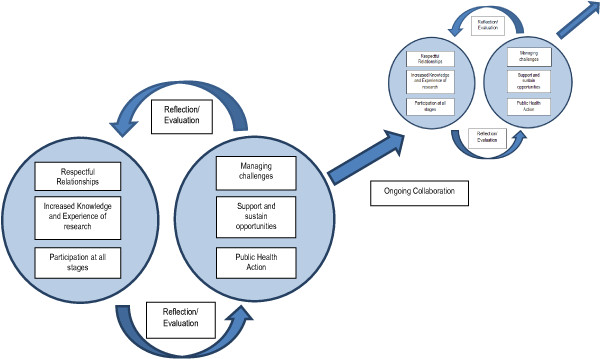
Elements of research capacity strengthening in the context of mutuality.

Five respondents chose face to face interviews facilitated by another member of the team (MRM or DM). Interviews were facilitated in both Solomon Islands Pijin (n=2) and English (n=3). Five respondents chose to provide written responses. All five were written in English. Interviews were transcribed and/or translated by a Pijin speaker. Written responses and interview transcripts were compiled in MS Word and imported into the qualitative software programme *NVivo 9.* Text was analysed and emergent themes identified using inductive grounded theory procedures [36]. Emergent themes were initially elicited by MRM and discussed with all authors. Necessary changes to themes were made in response to feedback. A visual model was created to conceptualise the linkages between emergent themes (Figure 1).

Ethics approval for the 2011 Health Research Workshop was granted by James Cook University Ethics Committee, Atoifi Adventist Hospital Administration Committee and the National Health Training and Research Institute, Ministry of Health and Medical Services, Solomon Islands.

## Results

Six major themes emerged from the interview and written responses: Respectful Relationships; Increased Knowledge and Experience of Research; Participation at all Stages of Research; Contribution to Public Health Action; Support and Sustain Research Opportunities; and Managing Challenges of Research Capacity Strengthening. These themes were common to both Solomon Islander and Australian respondents.

### Respectful relationships

Respectful relationships between research workshop facilitators, participants and East Kwaio community members were reported as pivotal to the success and sustainability of RCS activities. Research activities reportedly improved the often antagonistic relationship between AAH staff and the East Kwaio community, particularly people from the nearby mountain areas who have chosen not to convert to the introduced Christian religion and continue to practice ancestral religion. AAH staff are reportedly more respected by the East Kwaio community as a result of the workshop. One Atoifi researcher stated the RCS activities have *“created a good relationship between surrounding community and Atoifi hospital compared to our previous relationship with both the coastal and mountain community.”* Another Atoifi researcher reported *“Research is part and parcel of valuing things, people, community, public and being committed in doing the best for people through working together with them. We want to work very closely with mountain people to provide health services for them, as this will be an unique model which can’t be seen anywhere in the Solomons”.*

The reputation of AAH was perceived as being improved beyond the immediate community. RCS activities *“gives recognition to the hospital by the outside research institution like Solomon Islands Medical Training and Research Institute, Ministry of Health and JCU”,* stated an Atoifi researcher. One Australian researcher stated, *“There has to be an increase in reputation of Atoifi as a research place for doing research. The powerful bits about this project are that we are working with people that are incredibly influential in their community … they have influence in the area and also nationally”.*

Valuing community leaders as partners in research was identified as important for researchers from both Atoifi and Australia. For some this was a new way of working. One Australian (laboratory based) researcher stated*, “The approach is different to anything I have considered- I would not have considered people outside the scientific community and would have relied on local medicos- but community people are really interested in being part of the process.”* He went on to say, *“I thought it would be a mess but was proven wrong to my original assumptions. With local people we achieved more than I would have ever considered possible.”* An East Kwaio chief stated of his appreciation of chiefs and community members being involved.

“Hem mekim heart blo mi hem feel gud. Why mi feel gud, bikos, iumi involvem not only olgeta man save lo raetim, but disfela wokshop, samting mi hapi long hem nau, no mata man hem no raet, but, hemi putim tingting blong hem for iumi share together”.

Translation: It makes my heart feel good. Why I feel good, because, we involved not only the people who have knowledge to write, but this workshop, the thing I am happy with it, even if someone can’t write, but they could contribute their knowledge for us to share together.

Relationships between many of the Solomon Islander and Australian researchers have been ongoing for two decades (EK, HH, TRH, RA, DM & MRM) [19,20,37,38]. The respectful, sustained nature of these relationships has been critical to the research strengthening process. Respectful, mutual relationships were also important to the Australian researchers, as stated by one visiting Atoifi for the first time: *“Relationships were built in process- because we included people – not using an authoritarian outlook- we included people and mentored that is why we have built trusted relationships”.*

### Increased knowledge and experience of research process

Knowledge and experience of public health research processes were identified as key outcomes of the RCS activities. A number of researchers discussed the movement from theoretical to practical health research knowledge. *“Theory about research has become a reality as I practically apply what I have learnt in real practice. This has helped me to understand the meaning of the research terminologies and understand the whole process i.e. research hypothesis / question, various ways of data collection and analysis as well as compiling and reporting results,”* said one Atoifi researcher. An Australian (qualitative) researcher discussed the broadened understanding of research resulting from the workshop. *“I have learned more about research - my espoused methodology, the qualitative methods I employ for undertaking community based research and a whole new world of quantitative methods”.* Another Australian researcher reported, *“It (the workshop) has allowed me to have a greater understanding of the research process because it needed to be distilled into simple language to be accessible to all participants but still retain its research rigour”.*

Increased knowledge and experience of specific techniques used in qualitative research such as transcription of a face to face interview recording was identified by one chief with vast experience as an orator, community mediator and interviewer.

“We don’t know, hau bai iumi go about the process ia; taim mifala go… stori no moa; Iu laik repeatim olsem samting; iu laik long olsem, ah, lelebet part iu laik English moa long em; lelebet part iu laik language moa long em; lelebet part iu laik Pijin moa; and repeatim and repeatim; but mifala no save`, but taim word to word iu barava raetim samting ia…Man, ten minute interview or, twelve minute interview, iumi raetim about three hours. So datwan, man, hem wanfala samting wea mi barava learnem during long research workshop”.

Translation: We don’t know how to go about the process (of research); when we interviewed we repeated something, spoke a little in English, a little in Pijin, a little in language (East Kwaio), but when we wrote it word for word, man, a ten minute interview, or twelve minute interview you write (transcribe) for about three hours. So that, man, is something I really learnt during the workshop.

More confidence to undertake community based public health research was identified as an outcome of the workshop by researchers from both Solomon Islands and Australia. *“Why I can do research at any time is because of the confidence I gained after the workshop research experience in April,”* stated an Atoifi researcher. Another Atoifi colleague reported, *“I will start to look at priority areas that will need research and start with small projects. Firstly the small projects should give me enough confidence to start looking at major research projects”.* For Australian researchers, confidence to undertake research grew from being exposed to new people and new opportunities. One Australian reported, *“Testing out my capacity to be able to research in a team where I haven’t known anyone and build relationships two-ways in a short space of time in a respectful way. I have learnt, been able to go the journey with that and been enriched.”* Another Australian reported *“I have learnt about the potential for change if we just act together- no need to keep it tight and controlled- go with it using values as a basis for decision-making. If the community want it and we can contribute- let’s go”.*

The workshop elicited interest from nurses working in the hospital. *“A male registered nurse… was looking for a research book to further read on what was presented”* reported an Atoifi researcher. Nursing students were reported to be seeking involvement in research projects. *“A student asked for off day because she wants to go out and be involved in collecting data at the community as part of their practice and further asked for (a day) off on Thursday the second week to listen to the presentation, (it) is amazing”,* a Atoifi researcher reported. The researcher went on to explain that the knowledge and experience gained through the activities, *“will help me to teach others”.*

An increased understanding of the ethics of community based public health research was highlighted by researchers from both Atoifi and Australia. One Atoifi researcher stated, *“I must follow and abide by the ethics of research so that no negatives will interfere with the research process.”* For one Australian (laboratory based) researcher this was highlighted through the ethical consideration of respecting local cultural rules that meant faecal samples were not to be taken to Australia. The researcher stated, *“I would like to have taken samples back (to Australia) - I need to have value added”.* The “value” referred to was from the perspective of adding protozoal prevalence studies to the faecal samples in an Australian laboratory.

### Participation at all stages in research

Taking part in all stages of the research process was identified as important. One Atoifi researcher stated, *“actually taking part in the process or the stages that begins in drafting of proposal which involves consultation with the administration for consents/communities and individual …gives me the confidence to become motivated to undertake research”.* One Australian researcher reported, *“Some apparent challenges or difficulties were able to be overcome through leaders at the hospital and community participating in the workshop and that community leaders were able to assist in formulating the research question and methodologies – for example this was able to overcome some social and gender sensitivities around the collection and microscopy of human faeces for the gutworm study*”.

All respondents discussed the importance of the partnership with chiefs and community leaders, with one Australian stating the chiefs and community leaders’ participation had changed the way he thought about engaging participants in research.

“When we talked with (the chiefs) – if we had simply not had that talk, we wouldn’t have learnt anything about this (culturally-appropriate approaches to research). We would have thought local people were being difficult- it is a case of asking. As Daniel Borston said “the greatest obstacle is not ignorance of knowledge but the illusion of knowledge.” I would have planned with medicos I now know that if I do more research I will have the support of the people in the mountains. I would have gone in with colonial assumptions; I didn’t understand they were assumptions. I did a lot of learning about assumptions”.

Another Australian researchers stated that the chiefs, *“talked through the things they know about TB and which ones they would do (in the pilot study)…they chose TB in the mountains…it highlighted community feeling they owned it”.*

One East Kwaio chief reported how health research was typically not done in partnership with the people from Solomon Islands. *“Most of time, people think, research, hemi blong olketa white man, ol no save doem ia. …hem no fitim mifala olketa local man.”* Translation: Most of the time people think research belongs to the white men- they don’t know how to do it …its not something for us locals. He added,

“Mifela man no save read na raet…but still we got the wisdom, to share. So dat wan nao hem wanfala samting wei mi barava hapi long hem. Wei disfala workshop mi hapi long hem, nau dis wan, hem no wansait no moa, but everiwan, iumi insait long disfala research. And datwan hem makim mi barava hapi tumus”.

Translation: We (chiefs and community leaders from East Kwaio mountains) can’t read and write, but we still have wisdom to share. So that is one thing that makes me really happy. What I am happy about regarding this workshop is that it wasn’t just one group – but everyone - we were all included in the research. That makes me very happy.

Involvement of community chiefs and leaders ensured community participation in pilot research projects. An Atoifi researcher reported, *“This workshop opens the community’s mind to accept research work in their villages and not resisting it as before, because they have some knowledge about research”.* An Australian researcher revealed his surprise at “*the phenomenal response to requests for specimens that we achieved in the time we were here”,* demonstrating the willingness of community members to be involved.

### Contribution to public health action

The involvement of outside researchers, local health professionals and community members led to immediate public health action and wider influence on national public health policy. This was highlighted through three examples:

(i) Antehelmintic treatments for individuals and a whole village (Na’au, East Kwaio).

All people who had parasitic worm eggs identified in their faeces were offered anthelmintic treatment within 1 week. Since one of the study communities (Na’au) had a high prevalence of hookworm, AAH staff worked with the community and treated all residents using a mass drug administration with albendazole. One Atoifi researcher reported the experience of a chief who had participated in the pilot study.

“I was also approached by two villagers from Na’au, expressing how Albendazole relieved their long existing abdominal pain. They were complaining of this abdominal pain for almost over 10 years and was diagnosed of having peptic ulcer at the hospital, however after taking Albendazol during the de-worming process at Na’au it relieved their abdominal pain, this is a miracle to them and they were telling people about this experience”.

Village leaders led a community project to increase cleanliness including keeping pigs in designated areas and digging drains in low lying areas of the village. Within two weeks results from the parasite survey were included in an application led by community leaders to improve sanitation. *“Benefits were also the practical outcomes that have arisen because of the workshop… (it has) accelerated and provided supporting evidence for a sanitation project in the village”* (Australian researcher).

(ii) Screening for Lymphatic Filariasis of a whole village (Alasi, East Kwaio)

During the workshop, a male community member presented at Atoifi Hospital with elephantiasis, a clinical manifestation of lymphatic filariasis (LF). A survey was designed with input from Solomon Islander researchers and community members supported by the Australian researchers (primarily RS). Local ethics approval was obtained and an entire village survey conducted during one night (between 10 pm and 2 am) only one week after the clinical presentation. Samples were split with one set of blood slides screened at Atoifi laboratory and another sent to JCU (Australia) for antigen and antibody testing not available at Atoifi.

*“I was very impressed at the speed, skill and organisational ability of the Atoifi team to respond to the case by discussing the appropriate blood survey for lymphatic filariasis with LF experts in our team, engaging with community leaders to arrange the bleeding of the entire village in the middle of the night, training in appropriate technical aspects of collection, storage and transport of specimens and implementation of the whole study in a matter of days after the patient presented at the hospital”.* (Australian researcher)

The survey showed that LF transmission was not occurring in Alasi (manuscript in preparation). The results were reported back to the villagers in Alasi, with a now reduced anxiety about the likelihood of others developing elephantiasis as a result of LF. The result was also communicated to the Ministry of Health and assisted Solomon Islands to be granted LF-free status by WHO later in 2011.

(iii) Contribution to national health policy.

The RCS activities were also reported to be influencing other national and international health research agendas, despite the remote location of Atoifi and the limited resources. One Atoifi researcher reported the impact the TB pilot study had on the national research agenda.

“One successful story was the presentation of the TB survey at the National level by PHC (Public Health Co-ordinator) a few weeks ago…he was then interviewed and put to media. Now the National TB for the country are moving in to support the research project that have been started by Atoifi/JCU Team. (It has) resulted in everybody asking to know more about this research approach done by Atoifi and JCU”

### Support and sustain research opportunities

A number of new and expanded research activities at Atoifi were identified through the RCS activities, including expansion of TB, LF and intestinal worms research in East Kwaio (in both coastal and mountain regions) and support for non-communicable disease research such as studies on diabetes, tobacco and marijuana use. A number of village leaders in East Kwaio have expressed a strong desire to be involved in further studies in these areas.

The need to strengthen skills to support these opportunities including to report and publish research results was strongly identified. An Atoifi researcher explained, *“It’s the editing report writing that I see become the hiccough* (sic) *for me as this will give me the sense of achievement after all instead of having this negative thought of being not able to complete my own project. What I started I have the responsibility to complete”.*

The workshop was reported to have assisted the development of reporting skills for one Atoifi researcher.

“I have been working on a mini research project trying to understand what the textbook is saying when JCU came along to run the research workshop. All the questions I have been asking myself throughout the process have been cleared and answered during the two weeks training. As a result, I was able to complete the process of my mini-research project with a clear understanding on how I have done it. And this research will be presented at the 3rd biannual PSRH conference in Honiara on July 5–8, 2011. This will be my first step ever in presenting a research paper and I count it as a first-step in my professional development”.

An Australian researcher reported concern about sustaining ongoing research capacity building activities to enable improved health. *“The danger of present project is that it will not make changes to health services, (we) need to do further work to trial and make changes. It’s important the next steps are linked to health impact possibilities to improve health”.*

RCS undertaken at Atoifi, rather than having to leave the campus or the country, was identified as a benefit to both individual researchers and the institution. It allowed for strengthening of research knowledge and experience with a broad range of professional and community participants in the local context and using local examples. This allowed RCS while maintaining the operations of the hospital and college of nursing. It also encouraged researchers from Atoifi and Australia to consider postgraduate and other formal study. One Atoifi researcher said the workshop *“encourages me to do my PhD study in public health area”*. An Australian researcher discussed her plans to report learning from research capacity work in her PhD. Another Australian researcher expressed *“It would be wonderful to see ongoing exchanges of staff between Solomon Islands and Australia – both for formal training and short term exchanges”.*

### Managing challenges of research capacity strengthening

A number of challenges were identified throughout the workshop that required considered management. Understanding English was a challenge for some community leaders, most often during the formal presentations. Some JCU researchers gave presentations in Pijin (DM & MRM) but some presentations were given in simple English and periodically translated (or further explained) in Pijin by HH or Kwaio language by EK. This was not satisfactory, as reported by one chief.

“Taim olketa man blong iumi, long bush olketa kam down na participate, laik, Professor hem tok English. Taim hem tok English, for hem kam lo olgeta man e read na write, hem gud. But mifala man no save even English, but mifala no save, datwan hem barava olsem, hem barava, hem no gud long mifala”.

Translation: When our people from the bush came down to participate, like, the Professor spoke English. When he spoke English, for those who read and write, that is good. But for us who don’t know English, we don’t understand, that is very, like, its really not good for us.

Australian researchers agreed. One stated, *“I wished we could have had more Pijin/Kwaio used in the workshops as I am sure some of it was lost on the community participants, because they told me.”* Another reported, *“Language: when requesting feedback* (via written One Minute Reflections exercises at the end of each session) *we got asked really good research skills questions on some stuff we had spoken about, that we had said quite clearly in English. That was a negative”.*

Challenges to undertaking RCS were different depending upon the researchers’ country of origin and level of research experience. For Atoifi researchers challenges lay in the logistics of organising the workshop and how to encourage active participation in RCS activities while continuing to operate the Hospital and College of Nursing. One Atoifi researcher demonstrated this struggle, *“Nurses expressed their disappointment because they could hardly attend the research workshop - (it) is a clear indication of how they value this capacity building research workshop”.* The negatives for Australian researchers related to working in a less-resourced environment, including lack of electricity, printing, internet and other communication and health risks (malaria, gastroenteritis). However, the negatives were not insurmountable. One Australian identified ways forward, despite the challenge of limited infrastructure at Atoifi.

“Negatives include the difficulties in transport and communication – there is a lot of interaction and planning and follow up before and after the workshop. This is difficult when the internet and phone/fax are not working and there is only intermittent electricity. This makes collaborative writing of articles/manuscripts challenging and increases the amount of time required to finalise report writing – however reporting back to community through community forums at the village level does not face such issues and so results can get back quickly”.

The social expectations of having to perform duties as a senior researcher or *‘big man’* in the Solomon Islands and having many people in work and living spaces were also identified as challenges.

## Discussion

Responses from researchers and chiefs from Solomon Islands and visiting researchers from Australia demonstrate that *all* benefitted from the way this RCS activity was carried out. RCS was indeed two-way; it was a mutually beneficial experience. The RCS, however, was experienced differently for each researcher and community leader, but all researchers reported an increased capacity to undertake research. Although mutuality can be defined as “common to all parties” [11], in our experience, mutuality in RCS did not mean we all developed the same research skills. However, there was a common experience of enhanced ability - of increased confidence and capacity to undertake research as a result of the RCS activities and a desire to/for support further research at Atoifi and surrounding communities. This is consistent with research methodologies such as community-based participatory research and participatory action research which acknowledge the researcher/s *and* research participants (co-researchers) benefit from the research process [39-41].

From the emergent themes of our experience of RCS at Atoifi we created a visual model to conceptualise the linkages between the themes (Figure 1). This cyclical model encapsulates key elements buttressed by mutuality, including cycles of culturally inclusive reflection/evaluation by all groups involved in RCS activities. The elements of this model are all key to ongoing collaboration and further public health RCS at Atoifi.

### Mutuality and link to decolonising methodologies

Participating on different terms during AAH RCS included the acknowledgement that mutual needs were being met for all researchers. As Smith explains from neighbouring Aotearoa/New Zealand:

*“When indigenous people become the researchers and not merely the researched, the activity of research is transformed. Questions are framed differently, priorities are ranked differently, problems are defined differently, people participate on different terms *[35]*”.*

This is in contrast with the model of ‘expert’ researcher teaching the ‘learner’ researcher who is then ‘strengthened’. As described by Sherwood, “from such praxis the process becomes a two-way sharing and learning encounter that contributes to the building of valid and meaningful data” [42].

White privilege is acknowledged when working within a decolonising framework [35,43]. The acknowledgement of privilege for those of us who are the white Australian researchers working in Solomon Islands contributes to our ability to be open about the benefits we have received when undertaking RCS. There is also a level of privilege that occurs between those of us who are Solomon Islanders when we have the privilege of educational, religious and positional power. Ongoing antagonisms between the hospital staff (educated, Christian) and the traditionalist East Kwaio ‘bush people’ (illiterate, ancestor worship) are little-by-little being addressed to change this historical inequity. “Power redistribution is required for mutual, two-way RCS (research capacity strengthening), including access to information, language used, location of workshop, representation of outcomes, leadership of research processes” [41]. We have found there are mutual benefits for all researchers involved in RCS done in this way - the benefits flow two-ways. Being honest in claiming (Solomon Islanders) or devolving (Australian) historical power and being open to the possibilities of new approaches and models of working together brings many benefits for everyone.

### Our ongoing challenges

There are structural challenges in sustaining this type of RCS. Resources are not readily available for people-paced RCS. There are limited funding options for health RCS in Solomon Islands and limited incentives for supporting RCS from less resourced countries. Often heath research favours commercialised, profit building technologies in resource rich countries and does not strongly support RCS in small, remote or less resourced areas such as ours in Solomon Islands [44]. “They (research funding bodies) do not typically allocate funds for capacity-building or generate long-term relationships with research institutions in developing countries” [44]. However, progress is being made. Successful RCS activities at Atoifi, supported by a modest internal research grant from JCU and in-kind support by New South Wales Health have demonstrated the ability of Solomon Islander nurses and the associated local communities to undertake health research. This approach of RCS is now being shared at the national level, including a keynote presentation by RA, EK and MRM at the Inaugural Solomon Islands National Nurses’ Research Symposium in May, 2012 [29]. HH also presented about RCS conducted at Atoifi during a nurse educators training workshop in Honiara, the capital of Solomon Islands in 2012.

### How might this study benefit others?

The two-way benefits demonstrated through these RCS activities have led to direct public health action, not only within East Kwaio communities, but for national and international public health policy. This study lays a positive foundation for future public health research in East Kwaio and other areas in Solomon Islands. It provides a successful approach and model of key elements for RCS. Through this it demonstrates possibilities for researchers from less resourced and more resourced countries to address health research capacity, health inequity and support public health action.

### Sustaining research capacity strengthening

Sustaining two-way RCS activities, including implementing research plans is an ongoing challenge for Atoifi. As others have found previously, “RCS is a long term activity, requiring long-term investment—in the early stages, there is often little to show beyond the implementation of process” [41]. We are striving to sustain the initial steps with follow-up activities and greater Solomon Islands leadership in public health research at Atoifi. Following the workshop in 2011, further RCS activities have been undertaken at Atoifi in October 2011 and May 2012 with modest internal JCU funds and supported qin-kind by Atoifi Hospital and NSW Health. In response to a strong desire from local village leaders and community participants, two villages with a combined population of more than 350, were surveyed for intestinal parasites. A further round of interviews on TB management and HIV prevention were also undertaken, often led by health professionals or chiefs who participated in the 2011 workshop. Formal sessions also covered: further analysis of existing data; managing research data; scientific and grant writing; drafting manuscripts; and completion of research reports for publication. Numerous community leaders are now approaching Atoifi leaders requesting that their villages partner in public health research projects. This has informed a number of applications for research funds from national and international bodies.

As tangible benefits to communities are experienced and reported, these communities are acknowledging the practical value of health research and strongly support additional research that answers their local questions. Acknowledging the mutual nature of RCS and the benefits to local communities, Solomon Islander and international researchers will assist in the sustainability of RCS activities. This acknowledgement will also assist to address the power differences and shifts in relationship between players. It will also guide the dynamic relationships, capacities and skills of Solomon Islander and outsiders.

Following the 2011 workshop HH successfully applied for a Pacific Leadership Program – Greg Urwin Award and was hosted for a five month professional placement at the World Health Organization Collaborating Centre for Nursing and Midwifery Education and Research Capacity Building, and School of Public Health, Tropical Medicine and Rehabilitation Sciences at James Cook University in Australia. This program included research leadership activities in both Australia and Solomon Islands. This further strengthens the mutuality of RCS and bolsters the strong foundation for future research.

### Limitations to this study

Data collection for this paper was a mixture of written responses to questions (n=5) and face to face interviews (n=5). The face to face interviews were facilitated by two different researchers. This may have influenced the amount and type of data elicited from researchers involved. However, this respected cultural and gender norms and all participating researchers have reviewed this manuscript and there is consensus on our themes and analysis.

On analysing this data, we learnt much about the role of mutuality for RCS. We failed to ask a specific question about what researchers thought the benefits were for researchers from the other country, that is, we did not ask of Solomon Islanders what they thought the benefits of the RCS was for the Australians and vice versa. A specific question about the benefits for the ‘other group’ might have enhanced our understanding of the mutuality on the process.

This manuscript was drafted by an Australian researcher, reporting this ‘mutual’ experience. This epitomizes the unequal power, educational opportunity, language in which the publication is written and formal writing capacity that still lies with the most resourced, despite efforts to date. There is obviously a need for more time and resources to further develop independent writing and research reporting skills in those of us from Solomon Islands, as we have previously identified [24]. However all Solomon Islander authors collectively reviewed, critiqued, edited the draft manuscript until consensus was reached on the language and terminology used and themes and analysis of interview data.

## Conclusions

It is our experience that RCS can benefit both those historically labelled “experts” from more resourced countries and those being ‘strengthened’ in research skills from less resourced countries. We propose that RCS can and should evolve into a more open two-way, mutually beneficial process. When acknowledging the benefits for all parties involved in RCS done in this way, we begin to redistribute/reclaim power often held by the more resourced researchers. Respectful, mutual relationships, a shared knowledge and experience of research process and participation at all stages by all parties opens possibilities for improved health for the communities in which we work and for future health research activities.

## Competing interests

The authors declare there they have no competing interests.

## Authors’ contributions

MRM: Co-designed the research (including study tools), facilitated interviews with co-researchers, analysed the interview data, drafted and edited the manuscript. DM: Facilitated interviews with co-researchers, reviewed data analysis and edited manuscript. HH: Provided interview data, contributed to analysis of data and edited manuscript. RA: Provided interview data, contributed to analysis of data and edited manuscript. RTH: Provided interview data, contributed to analysis of data and edited manuscript. EK: Provided interview data, contributed to analysis of data and edited manuscript. RS: Conceived the concept of study, co-designed the research (including study tools), reviewed data analysis and edited manuscript. All authors agree with manuscript results and conclusions.
